# Understanding the physicochemical properties of Zn–Fe LDH nanostructure as sorbent material for removing of anionic and cationic dyes mixture

**DOI:** 10.1038/s41598-021-00437-w

**Published:** 2021-11-01

**Authors:** Rehab K. Mahmoud, Mohamed Taha, Amal Zaher, Rafat M. Amin

**Affiliations:** 1 Department of Chemistry, Faculty of Science, Beni-Suef University, Beni-Suef, 62511 Egypt; 2 Materials Science and Nanotechnology Department, Faculty of Postgraduate Studies for Advanced Sciences (PSAS), Beni-Suef University, Beni-Suef, Egypt; 3 Environmental Science and Industrial Development Department, Faculty of Postgraduate Studies for Advanced Sciences, Beni-Suef University, Beni-Suef, Egypt; 4 Department of Physics, Faculty of Science, Beni-Suef University, Beni-Suef, 62511 Egypt

**Keywords:** Chemistry, Materials science, Physics

## Abstract

In our work, the removal of cationic and anionic dyes from water was estimated both experimentally and computationally. We check the selectivity of the adsorbent, Zn–Fe layered double hydroxide (LDH) toward three dyes. The physical and chemical properties of the synthesis adsorbent before and after the adsorption process were investigated using X-ray photoelectron spectroscopy, energy dispersive X-ray, X-ray diffraction, FT-IR, HRTEM, and FESEM analysis, particle size, zeta potential, optical and electric properties were estimated. The effect of pH on the adsorption process was estimated. The chemical stability was investigated at pH 4. Monte Carlo simulations were achieved to understand the mechanism of the adsorption process and calculate the adsorption energies. Single dye adsorption tests revealed that Zn–Fe LDH effectively takes up anionic methyl orange (MO) more than the cationic dyes methylene blue (MB) and malachite green (MG). From MO/MB/MG mixture experiments, LDH selectively adsorbed in the following order: MO > MB > MG. The adsorption capacity of a single dye solution was 230.68, 133.29, and 57.34 mg/g for MO, MB, and MG, respectively; for the ternary solution, the adsorption capacity was 217.97, 93.122, and 49.57 mg/g for MO, MB, and MG, respectively. Zn–Fe LDH was also used as a photocatalyst, giving 92.2% and 84.7% degradation at concentrations of 10 and 20 mg/L, respectively. For visible radiation, the Zn–Fe LDH showed no activity.

## Introduction

The textile industry is characterized by the consumption of large quantities of water, much of which contains dyes^1^. There are many industries that use dyes, such as the paper, plastics, and leather tanning industries^2^. The effluent discharge of the textile industry leads to environmental pollution owing to the existence of complex mixtures of methylene green (MG), methyl orange (MO), and malachite blue (MB) as cationic and anionic dyes^3^ and toxic metal ions in polluted water^4^. The usage of dyes has a hazardous effect on all life forms, that is, humans, plants, and animals; therefore, their effective disposal is necessary^5^. Thus, different chemical and physical techniques have been applied, such as biodegradation, reverse osmosis, activated sludge, chemical oxidation, and electrochemical methods involving membrane separation, chemical oxidation, anaerobic and aerobic microbial degradation, adsorption, and photodegradation^6–8^.

The adsorption process is thought to be more efficient when compared with other physicochemical wastewater treatment techniques^8–12^. Activated carbon, which is widely used as an adsorbent for wastewater treatment, is expensive and therefore, uneconomical^13^. The removal of organic pollutants with the use of layered double hydroxide (LDH) has been of great interest to many researchers in recent years. This is because of its unique features and properties, such as high surface area, low toxicity, low cost, high capacity of anion substitution, recoverability, and high stabilities for chemical and thermal properties^14^. Several techniques have been reported for the modification of LDHs, for example, the reconstruction process, the ion exchange process, and coprecipitation in the presence of organics^15^. Many limitations of LDHs remain, including the inability to used in highly acidic or basic medium. The challenge is the preparation of LDH materials by applying new techniques and using advanced modifications, environmentally friendly methods, and easy operation. In our study, we selected Zn–Fe LDH as a model over other LDHs owing to its high stability constant at nearly 25.27 and low solubility product reach of 62.51^16^. Furthermore, increasing amounts of solid adsorbent wastes require the development of new recycling methods. This is a critical requirement around the world^17^.

Many gaps still exist in the science describing the flow of electrically conducting fluids, and such gaps are most regular with regard to multiphase fluids (i.e., nanofluids). Finally, despite the availability of many environmental applications, to date there are few to no reported environmental or medical applications involving nano-conducting fluids. With increasing investigation, it is predictable that nanofluids can make a considerable impact in many applications. So, the dielectric behaviour of solid materials has been reported and explained using different models. The purpose of this study is to provide an overall account of the dielectric properties of a material. Dielectric studies could also aid an understanding at the molecular level of the basic interaction of the nanoparticles in aqueous systems^18^. The study of the characteristic optical properties of a material is important in providing data regarding electronic transitions, fundamental gaps, localized states, and trapping levels. Absorption of visible light from the top of the valence band (which is mainly composed of oxygen (O) 2p orbitals hybridized with Fe or Zn 3d orbitals) to the bottom of the conduction band (which is mainly composed of Fe or Zn 3d orbitals hybridized with O 2p orbitals) is the reason for the electronic band gap transition^19^.

**Figure 1:**
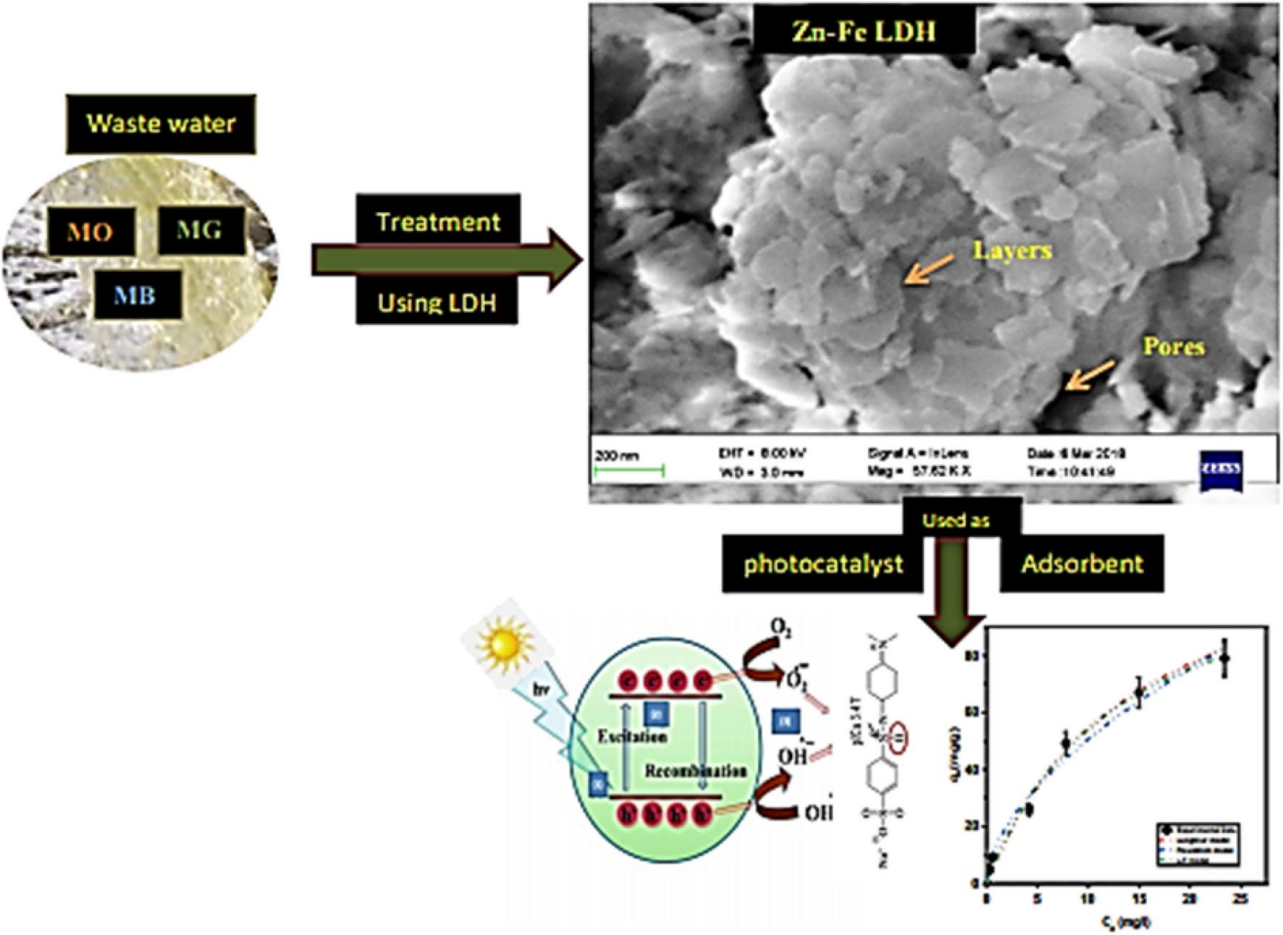
The proposed application performed using Zn–Fe LDH.

The process of degradation has been recommended as an effective environmental strategy for remediating organic pollutants such as dyes, especially when using low-cost semiconducting metal oxides as photocatalysts^20,21^. Therefore, after an experimental study of the adsorption process, we examined the LDH applied as a photocatalyst for the MO dye. In this study, we aim to analyze a multiadsorbate system by studying the selectivity of the main dye in the ternary system and then the interaction and behavior of two model cationic dyes (MB and MG) and one model anionic dye, MO^22^, with Zn–Fe LDH applied as an effective adsorbent material. The Zn–Fe LDH prepared was well characterized by FT-IR, XRD, FESEM, HRTEM, UV–Vis spectroscopy, N_2_ adsorption/desorption, zeta potential, partial size analysis, and XPS. The adsorption mechanism of the electrical behavior was analyzed. This study highlights the potential application of Zn–Fe LDH as an efficient adsorbent of anionic and cationic dyes and its electrical properties, which extend its scope for application in environmental remediation processes (Fig. 1).

## Experimental details

### Materials

Zn(NO_3_)_2_·6H_2_O was purchased from Chem-Lab NV, Belgium, Fe(NO_3_)_3_·9H_2_O. Hydrochloric acid was supplied by Carlo Erba reagent while NaOH was supplied from Piochem for laboratory chemicals, EGYPT. MB, MO, and MG powder were purchased from Oxford Laboratory Reagents (India) Table S1. All the mentioned chemicals have been used without any purification. The experiments and preparation of the material were performed using deionized water, which is free from CO_2_.

### Synthesis of Zn–Fe LDH

In our work, Co-precipitation method was used to prepare Zn–Fe LDH. The added solution of Zinc and iron as nitrate precursors by 4:1 molar ratio (Fig. S1a). Slow flow rate of 0.10 mL/min of NaOH (2 M) solution was added till pH 10 for complete the precipitation. The resulting material was aged and kept at 60 ± 0.5 °C for 12 h and then was filtered and washed several times using distilled water to get rid of excess OH^−^ and then washed using ethanol. Finally, the adsorbent sample was dried at 80 ± 0.5 °C for 24 h^23^.

### Characterizations of the prepared material

The formed LDH/nitrate type has been characterized by XRD (PANalytical Empyean, Sweden). The accelerating voltage used was 40 kV, 30 mA current, ranging from 5° to 60° scan angle, and scan step of 0.05°. To determine the vibration of chemical bonds, Bruker (vertex 70 FTIR-FT Raman) Germany spectrophotometry (serial number 1341) covering a frequency range of 400–4000 cm^−1^ has been applied and used potassium bromide disc. The morphology of materials has been estimated by Field Emission Scanning Electron Microscope (FESEM) Germany. The EDAX (Quanta FEG250, Germany) had been used to determine the molar ratio of Zn–Fe LDH. The BET specific pore volume, specific surface area, and pore size distribution of the nano-adsorbents were determined by N_2_ adsorption using an automatic surface analyzer (TriStar II 3020, Micrometrics, USA). For analyzing the elemental composition of the prepared material (Kratos-England), X-ray photo electron spectroscopy (XPS) with Al-KαX-ray mono chromatic source (hυ = 1486.6 eV) has been used. Zeta potential and hydrodynamic particle size were investigated by te Nano-Zeta sizer (Malvern Instruments Ltd, United Kingdom). Using high-resolution transmission electron microscopy (HRTEM, JEOL-JEM 2100) to determine the microstructures of the used LDH. The procedure of sample preparation for zeta potential measurements was as explained in our previous work^14^

### Investigation of optical properties

The optical band gap of the sample material was performed with the Kubelka–Munk (K–M) function using the following equation^24^:

1 $$F(R) = \frac{{(1 - R)^{2} }}{2R} = \frac{K(\lambda )}{{S(\lambda )}},$$

where *F*(*R*), *R*, *K*(*λ*), and *S*(*λ*) are the K–M or re-emission functions, the diffuse reflectance of the sample, the absorption coefficient, and the scattering coefficient, respectively.

The absorption coefficient α was calculated using the Lambert Law from the measured absorbance results ^25,26^:

2 $$\alpha = 2.303A{/}d.$$

Where A : the optical absorbance and d: the sample thickness. The following expression, suggested by Tauc, Davis, and Mott, is used:

3 $$(\alpha h\nu )^{1/n} = A(h\nu - E_{g} ).$$

where h,ν, hν and C are the plank's constant, the frequency, the incident photon energy, and proportional constant, respectively. E_g_(eV) is the band gap energy of the material, and the index n determines the kind of transition. It can be equal to 1/2, 2, 3/2, or 3 for directly allowed, indirectly allowed, forbidden direct, and forbidden indirect transitions, respectively. In this case of direct transitions of Zn–Fe LDH nanoparticles, the value of *n* is equal to 1/2.

The acquired diffuse reflectance spectrum is converted to the Kubelka–Munk function. The vertical axis is then converted to the quantity *F*(*R*_*∞*_), which is proportional to α. The α is substituted by *F*(*R*_*∞*_) in the Tauc equation. Thus, the relational expression in the experiment becomes

4 $$\left( {h\nu F(R_{\infty } )} \right)^{2} = A(h\nu - E_{g} ).$$

### Dielectric properties

The dielectric behaviour of Zn–Fe LDH samples as a function of frequency was studied in the form of the dielectric constant, dielectric loss, and the ac conductivity (*σ*_*ac*_) at different temperatures, including the effect of gamma irradiation on it. The dielectric properties of the nanoparticles are studied using a HIOKI 3532 LCR HI-TESTER in the frequency region from 200 Hz to 5 MHz. The nanoparticles are made into pellets, and the surfaces of the samples were coated with a silver paste and placed between the two copper electrodes that act as a parallel plate condenser.

The dielectric constant (*ε′*) of the material is measured by using the formula

5 $$\varepsilon^{\prime} = \frac{Cd}{{A\varepsilon_{0} }}.$$

where C is the capacitance, *d* is the thickness, *ε*_0_ is the free space permittivity and *A* is the area.

### Adsorption study

Several experiments were performed to obtain data regarding the influence of the solution pH, adsorbent amount, initial dye concentration, and the selectivity of LDH toward applied dyes. Falcon tubes (50 mL) contained 0.05 g of the synthesized adsorbent and 20 ppm of dye as a pollutant. The pH of the dye solution was adjusted from 3–10 using HCl or NaOH (0.10 N), and measurements were made with a Metrohm 751 Titrino pH meter. The adsorption steps were performed for two other dyes. All experiments took place in the dark, and the Falcon tubes were put on an orbital shaker (SO330-Pro) for 20 h at 250 rpm until reaching equilibrium. A UV–Vis spectrophotometer (UV-2600, Shimadz, Japan) was used to estimate the residual concentration of each dye at a wavelength of 675, 464, and 618 nm for MB, MO, and MG, respectively^27^. To check reproducibility, all experiments were performed in triplicate. Upon estimating the pH of each dye, a pH value of 9 was estimated for MB, 6 for MO, and 5.4 for MG; and the effect of the initial dye concentrations was determined for each dye by adding 0.05 g of catalyst to 50 mL of the dye solution. After each adsorption process, syringe filters (Millipore Millex-G, 0.22 µm pore size) were used to separate the catalyst from the solution. Uptake experiments were performed in batch mode to estimate the effects of the initial concentration of MB and the other competing dyes (MO and MG). The amount of dye removed is estimated by:

6 $$q_{e} = (C_{o} - C_{t} ) \times V/W$$

7 $$\% Q = (C_{o} - C_{t} ) \times 100/C_{o} .$$

Equilibrium conditions were investigated by isotherm models and discussed in terms of nonlinear equations. We demonstrated the significance of our results using the statistical parameters R^2^ and χ^2^:

8 $$\chi^{2} = \sum (q\exp - qcal)^{2} {/}qcal^{2} .$$

Sun-light driven photocatalytic dye degradation was applied in the experiments. The LED visible source was a lamp from Philips model 3PM5 with 14 W of nominal power. No cut-off filters were used for irradiation. The photodegradation of methyl orange dye was performed by using a photocatalytic glass reactor containing of a cylindrical glass tube. The experiments were happened in sunlight between 11 am to 3 pm when the sunlight intensity had been nearly constant with low variation. The experimental procedures of the included degradation tests were done by mixing definite amounts of the LDH as a photocatalyst with MO dye solution in the dark for about 24 h as a step to achieve the dye adsorption/desorption equilibrium state. After that, the photocatalytic activity happened in the visible light. After adjusting the test volume to about 50 mL and the reaction temperature to 35 °C, the photodegradation parameters (dosage (10 mg), concentration (10 and 20 mg/L), pH (pH 8), and contact time (to 240 min) were determined. At the end of the experiment, LDH particles were separated from the solutions by centrifugation and the residual concentrations of MO dyes was estimated.

The chemical stability of Zn–Fe LDH was performed using 0.10 g of adsorbent and was added into 200-mL aqueous solution at different initial pH (2.5–11) and shack for 24 h. Then, the dissolved Zn^2+^ ions in the solution were detected by using an atomic absorption spectrophotometer (model ZEISS-AA55, Germany). Also, to investigate the chemical stability of adsorbents, XRD spectra were investigated after the adsorbents were collected and dried in a dryer at 60 °C.

### Consistency of results and quality assurance

The remaining concentration of the dye in samples was recorded using a UV–Vis spectrophotometer. The plastic and glassware used in the research experiments were cleaned and washed with 5% HCl aqueous solution and then immersed in bidistilled water. All chemicals used in the research experiments were of high grade, and the precision in dye records was determined by consecutively inserting each dye solution standard into the UV–Vis spectrophotometer to get a calibration curve (R^2^ = 0.999). After every 15 samples, 3 standard solutions of dye were run to confirm the reliability of data from the spectrophotometer. All experiments were performed in triplicate to ascertain reproducibility, and the average concentration was estimated by applying the mean and standard deviation (± SD) obtained from SPSS version 16. A p-value of less than 0.05 was taken to be statistically significant.

### Monte Carlo (MC) simulation

The MC simulation was performed by the Adsorption Locator module as implemented in the BIOVIA Materials Studio 2017 package (https://www.3ds.com/products-services/biovia/products/molecular-modeling-simulation/biovia-materials-studio/). The Zn–Fe LDH models were built from the crystal structure of hydrotalcite [Mg_3_Al(OH)_8_]. The Mg^2+^ and Al^3+^cations were replaced by Zn^2+^ and Fe^3+^ cations, respectively. The cell formula was Zn_20_Fe_5_ (OH)_50_(NO_3_)_5_, and the cations distribution was adopted as reported by Fan et al.^28^ for the 4 (M^2+^/M^3+^) molar ratio. The cell and the studied dyes were optimized using the Universal forcefield^43^, and the QEq charge method^29^ was applied. The optimization process was done by the Forcite module, as implemented in the Materials Studio 2017 package. The convergence tolerance quality was set to be ultra-fine.

**Figure 2:**
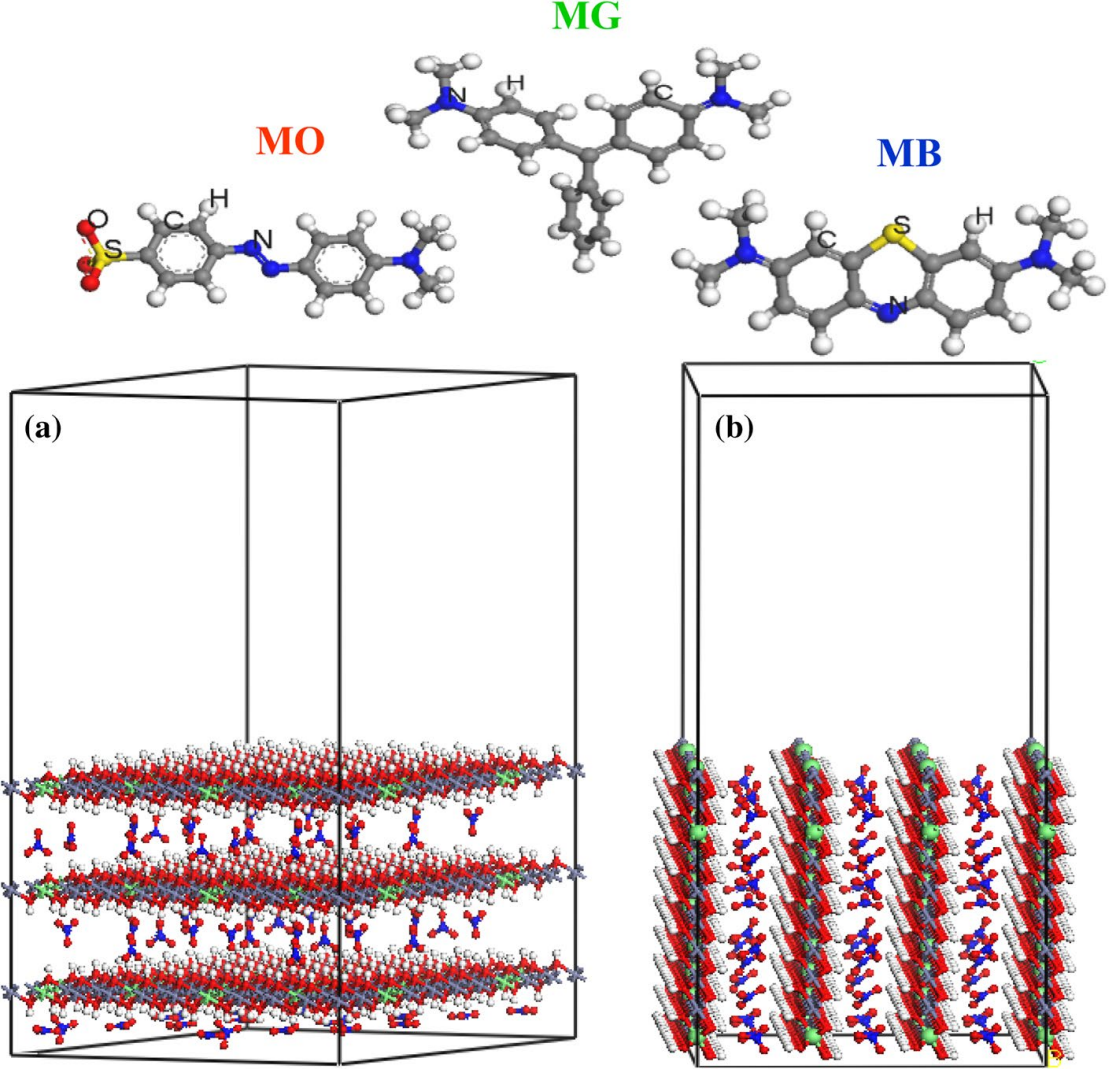
The optimized dye molecules and (**a**) Zn–Fe LDH (0 0 1) and (**b**) Zn–Fe LDH (0 1 0) surfaces; Fe atoms (cyan), Zn atoms (violet), O atoms (red), N atoms (blue), H atoms (white).

The adsorption of MO, MB and MG molecules on the Zn–Fe LDH surface was carried out using MC simulation, by using the adsorption locator module that uses the Metropolis MC method to obtain the lowest-energy conformers between the adsorbate and adsorbent surface. This module calculates (Δ*E*_ads_). Two surfaces were cleaved from the optimized constructed cell using build tool in the Materails Studio package, that is, LDH (001) and (010) surfaces. A 35 Å-thick vacuum slab above the LDH surfaces was created, and the two models are shown Fig. 2. As shown in this figure, the (001) and (010) planes represent the hydroxyl, and Zn–Fe LDH facets, respectively. The van der Waals force and electrostatic interaction were handled by the atom-based and Ewald methods, respectively.

## Results and discussion

### Material characterization

**Figure 3:**
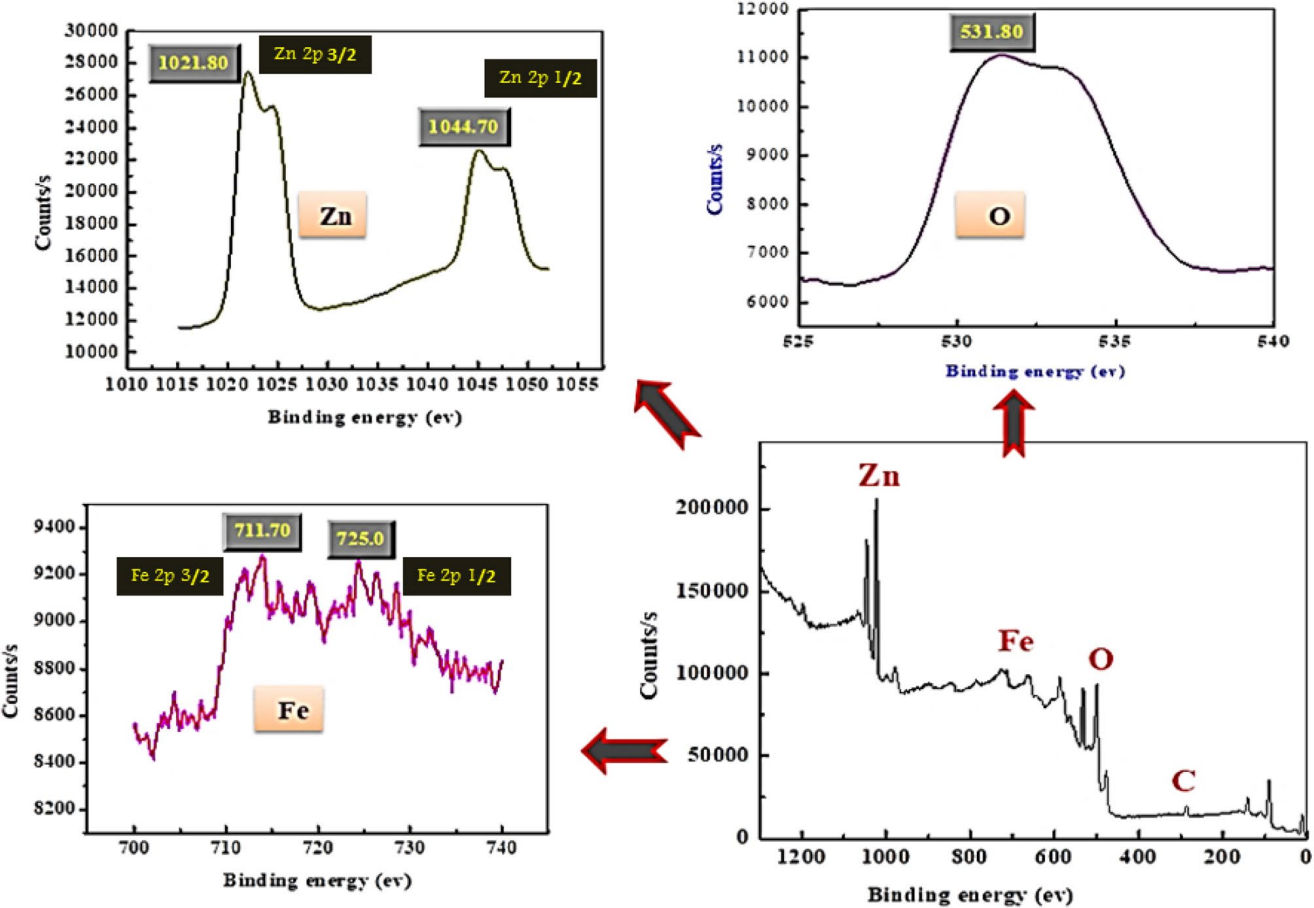
X-ray photoelectron spectroscopy spectra of the prepared Zn–Fe LDH.

FESEM images were applied to perform the morphology of the synthesis LDH as shown in Fig. S1b–d. It displayed the characteristic sheets, layers, and hexagonal like morphology of LDH. Using precipitation technique for adsorbent preparation may be the reason of the layers accumulation in this statue and also the reaction rate and time have been an important role on the thickness and shape of prepared sample^23^. It could be due to the slow nucleation for precipitate formation^30^. EDX analysis was performed to confirm the presence of Zn, Fe and O as seen in Fig. S1a. As shown in Fig. S2a–d (HRTEM) images show uniform hexagonal and layers structure of Zn–Fe LDH and confirms the polycrystalline nature^31^. The Surface property of synthesized LDH are followed to type II isotherm that matches with H_3_-type hysteresis loop and that is related to the mesopores/macroporous construction and capillary condensation process. The prepared sample has surface properties that estimated by (BET) method. BET surface area, the total pore volume, and average pore size of the sample are 71.61 m^2^/g, 0.078 cm^3^/g, and 2.61 nm, respectively. The average pore size is < 50 nm and there is extensive spreading of pore size up to 16 nm (Fig. S2h). (XPS) was applied for analysis the composition of LDH and assure the bonding of containing atoms. As observed from (Fig. 3) the XPS spectrum proved the existence of Fe, O, and Zn. The Zn 2p spectra of XPS displayed two peaks asymmetric assigned to Zn 2p 3/2 and Zn 2p 1/2 core levels at 1021.8 and 1044.7 eV respectively, which related to that Zn charge being in 2+ oxidation state in LDH^14^. The O 1s peak as shown in Fig. 3 of pure material (LDH) was at 531.80 eV which due to the presence of the -OH group of Zn–Fe LDH^32^. Moreover, the signal of Fe 2p of Fe^3+^ peaks intricate by Fe 2p3/2 (711.7 eV) and Fe 2p1/2 (725.5 eV) refer to small positive change^33^.

The spectra of FTIR and XRD for Zn–Fe nitrate LDH were presented in Fig. S3 and confirm the structure of Zn–Fe LDH as discussed previously^34^.

**Figure 4:**
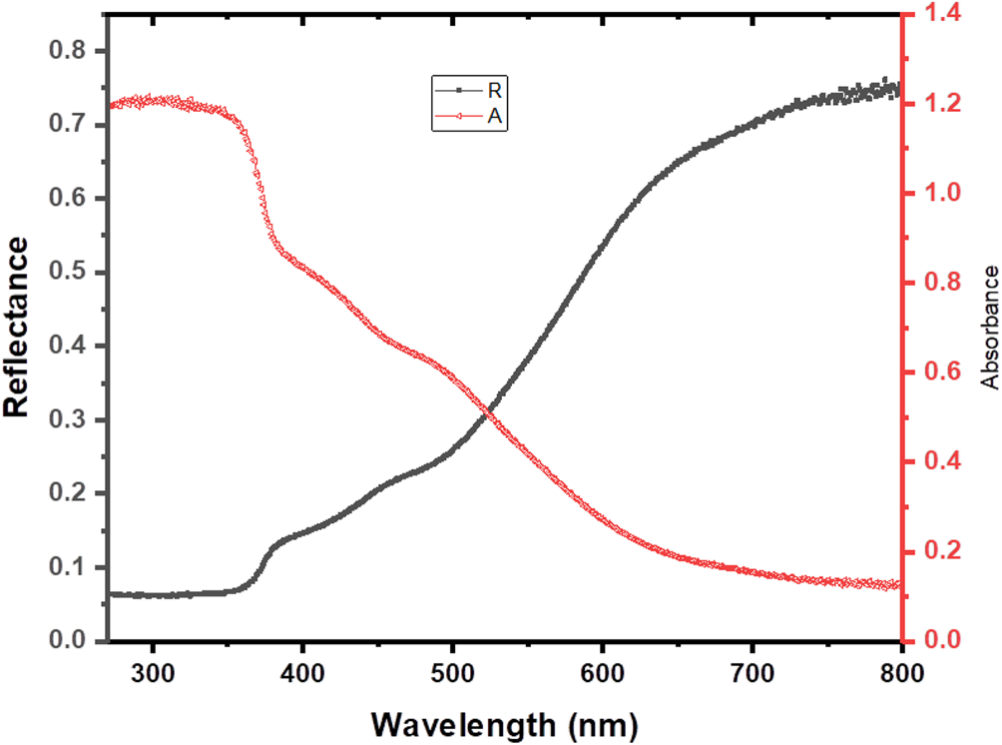
UV–vis reflectance and absorbance of Zn–Fe LDH.

**Figure 5:**
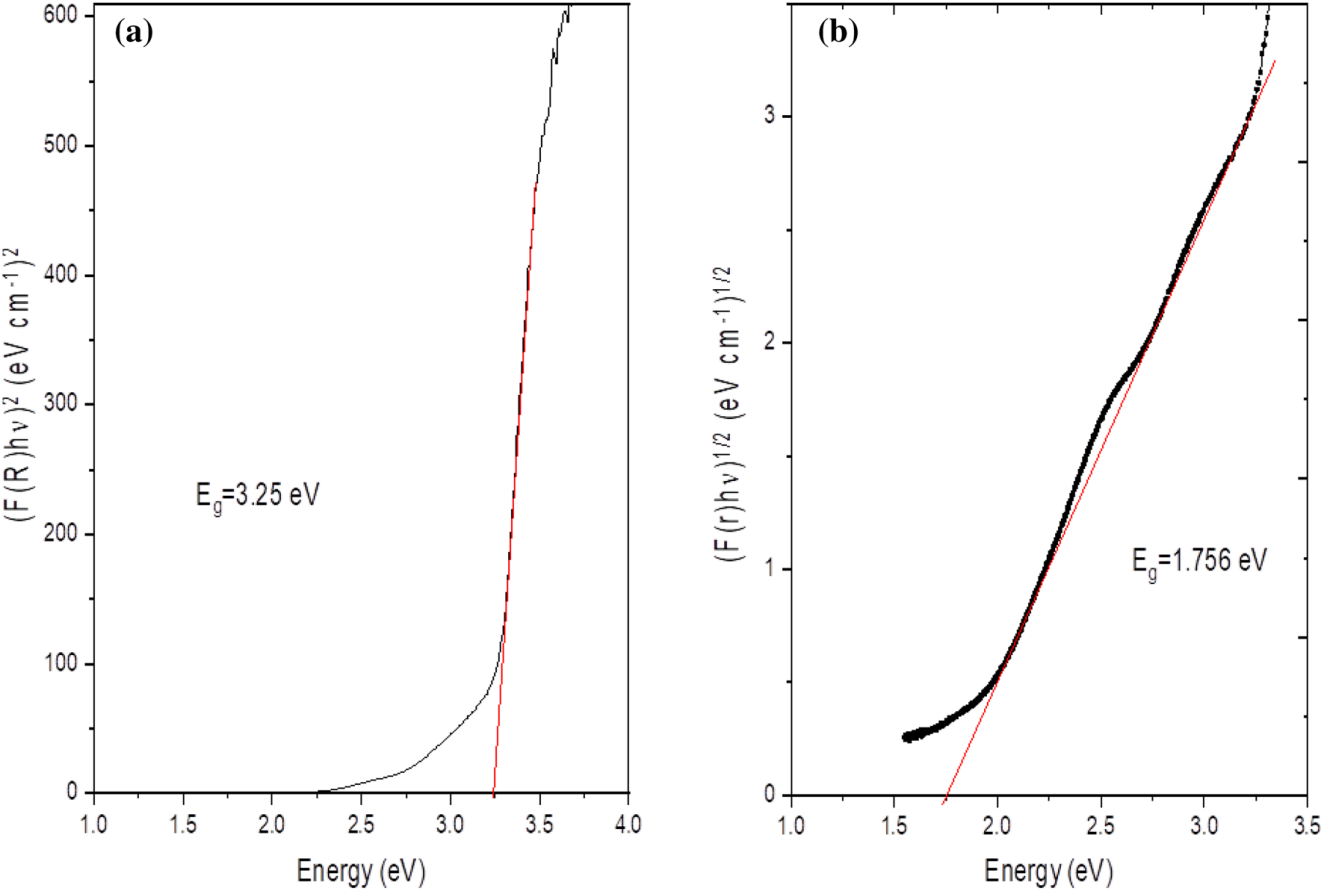
Tauc plot showing a possible fit to obtain the bandgap for Zn–Fe LDH with (**a**) direct and (**b**) indirect transitions.

The optical or photon properties of Zn–Fe LDH samples, such as the band gap energy, were identified using UV–Vis (NIR spectroscopy, DRS), and the resulting spectrum is displayed in Fig. 4, which describes the diffuse reflectance spectra of the samples. The average reflectance of the samples can be quantitatively expressed as an integral of the wavelength diffuse reflectance spectrum at the limits of 200–800 nm. The (*hνF(R*_*∞*_))^2^ was drawn against the *hν* using the Kubelka–Munk function, and the direct band gap of Zn–Fe LDH nanoparticles could be evaluated by extrapolating the linear part of the curve as shown in Fig. 5a. We observed that the linear part of the graph confirms the direct band gap-type behaviour of Zn–Fe LDH nanoparticles, and the approximate direct optical energy band gap is 3.25 eV. The observed absorption coefficient value (*α* < 10^4^ cm^−1^) is representative of the indirect band gap for Zn–Fe LDH as a function of photon energy (*hυ*). We plot (*αhυ*)^1/2^ and extrapolate the linear portion of curves to the values of (*αhυ*)^1/2^ = 0. The intercepts in Fig. 5b give the value of the indirectly allowed band gap energy (*E*_*g*_ = 1.76 eV).

The theory of reflectivity of light has been used to calculate the values of other optical parameters like *k* and *n*. The values of *k* and *n* have been calculated using the following equations^4–6^:

9 $$k = \alpha \lambda /4\pi$$

and *n* = (*1* + *R*) + [(*1* + *R*)^2^* − *(1* − R*)^2^(*1* + *k*^2^)]^1/2^/(*1 − R*).

**Figure 6:**
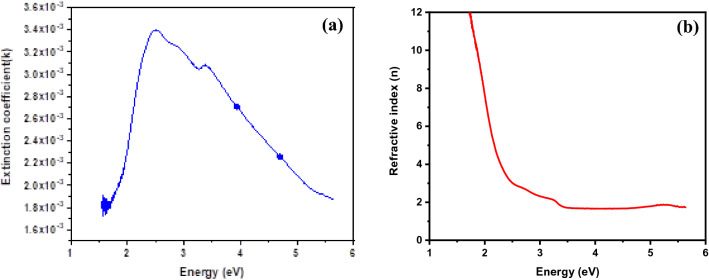
(**a**) Variations in the extinction coefficient (*k*) and (**b**) variations in the refractive index (*n*) as a function of energy for Zn–Fe LDH.

The variances of the refractive index and the coefficient of extinction with energy are shown in Fig. 6a,b, respectively. The extinction coefficient is an indicator of the amount of energy lost in the substance owing to the dispersion or absorption caused by molecules and particles. The extinction coefficient is high in the 200–360 nm wavelength range and low in the 360–800 nm wavelength range. The changes in the coefficient of extinction are related directly to the absorption of light, whereas the value of the refractive index decreases sharply with an increase in the photon energy in the visible region up to *λ* = 500 nm; it is almost constant in the 500–800 nm region. For the additional investigation of the optical data, several useful relationships can be inferred to link the real and imaginary parts of the dielectric function and the optical constants (*n* and *k*). The accompanying relationships have been utilised to compute the values of the real part (*ε*_*r*_) and imaginary part (*ε*_*i*_) of the dielectric constant for Zn–Fe LDH^4^

$$\varepsilon_{r} = n^{2} - \kappa^{2} \quad {\text{and}}\quad \varepsilon_{i} = 2n\kappa .$$

**Figure 7:**
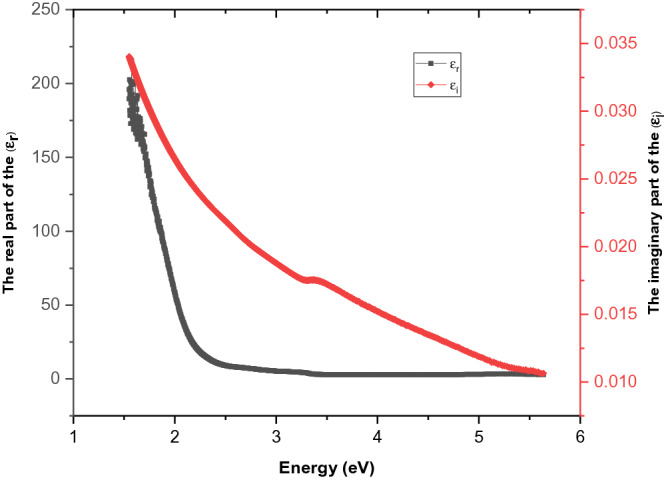
Variation of real and imaginary parts of the dielectric constant with incident photon energy (*hν*).

Its variety with photon energy is depicted in Fig. 7. From Fig. 7, we can see that the dielectric loss and dielectric constant diminish with photon energy analogues according to the behavior reported in the literature^35^. The magnitudes of the real dielectric constant are higher than the imaginary dielectric constant since they are reliant on *n* and *k* values. The real part of the dielectric constant contains a term that describes the amount by which it will impede the speed of light in the material, and the imaginary part shows how a dielectric absorbs energy from an electric field because of dipole movements^36^.

**Figure 8:**
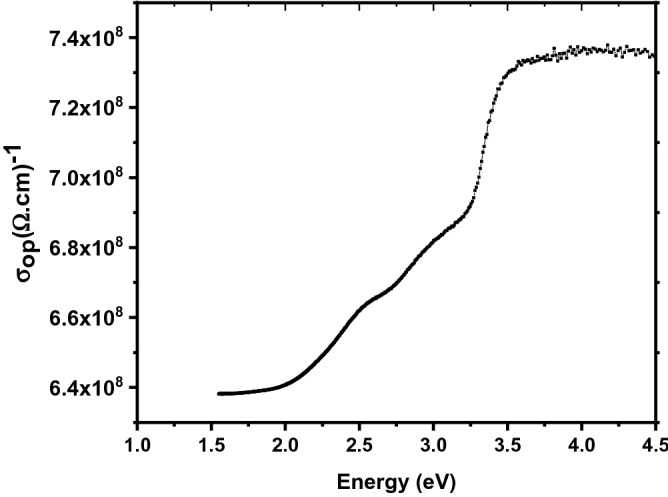
Optical conductivity as a function of photon energy.

The optical conductivity, which is related to the refractive index and absorption coefficient as given below, is then determined:

$$\sigma_{opt} = nc\alpha /4\pi ,$$

where *c* is the speed of light in a vacuum. The reliance of the optical conductivity on the incident photon energy for various Zn–Fe LDH nanoparticles is displayed in Fig. 8. It can be observed that absorption is moderately low at high wavelengths, indicating a lower probability of electron transition to higher energy bands. On the contrary, at low wavelengths (i.e., at high energies), absorption is high, demonstrating greater opportunities for electron transitions.

**Figure 9:**
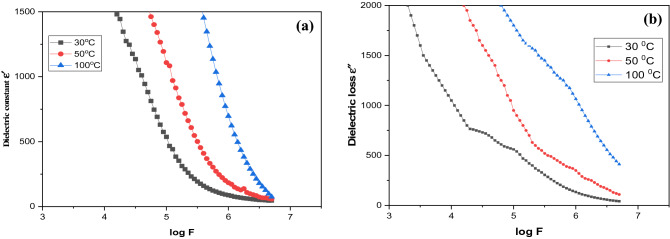
(**a**) The dielectric constant and (**b**) the dielectric loss as a function of frequency at different temperatures.

Figure 9a outlines the change in the dielectric constant ($\varepsilon^{\prime}$) for samples to the frequency at various temperatures. The dielectric constant $\varepsilon^{\prime}$ is a measure of the stored charge. The following clarification may be given regarding the nature of the dielectric permittivity for free oscillating dipoles in a varied field. When ω << τ^−1^, then *ε′* = εs, and dipoles follow the field. Dipoles start to lag behind the field as the frequency increases (with ω < 1/τ), and *ε′* decreases slightly. The dielectric constant drops (relaxation process) when the frequency surpasses the characteristic frequency (*ω* = *1/τ*). Dipoles do not comply with the field at this point,and *ε′* = *ε*_*∞*_(high frequency values of *ε′*) at extremely high frequencies (ω >>> 1/τ). At low frequency, the dielectric constant is very high, and it is initially found to diminish with frequency and then to become somewhat stabilized. The high value of *ε′* at frequencies less than 1 kHz, which increases as the frequency diminishes and the temperature increases, corresponds to the system's bulk effect. The issue of interfacial charge carriers is an important factor for the improvement of dielectric values in the frequency region. The requirement for a high value of the dielectric constant in the low-frequency area can obstruct the charge carriers at the electrode. At low frequency, the dielectric loss is extremely high, but with increasing frequency, it falls rapidly. The dielectric loss increases with increasing temperature, analogous to the temperature reliance of the dielectric constant as shown in Fig. 9b. At chosen frequencies, the dielectric loss value is found to increase as a function of temperature. This mechanism can be joined with the lagging behind of charged ion species with the applied energy that prompts polarization.

**Figure 10:**
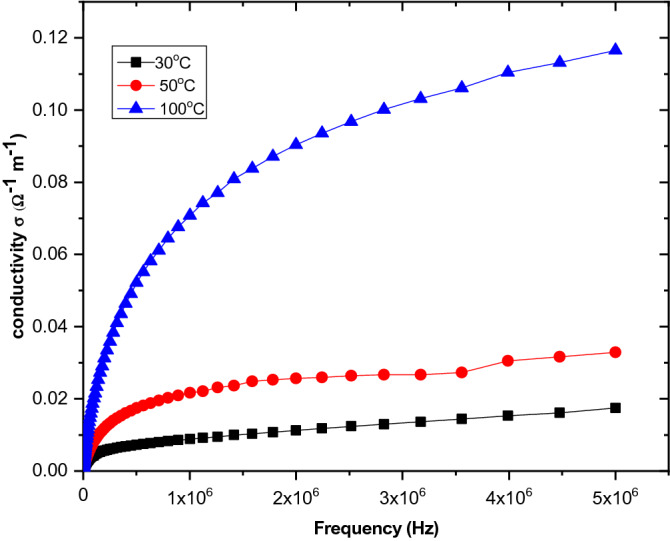
Frequency dependence of AC conductivity of Zn–Fe LDH at different temperatures.

Figure 10 shows the variety of AC electrical conductivity σ_ac_ of Zn–Fe LDH as a function of frequency at various temperatures. The conductivity plot has the accompanying characteristics: (i) scattering at lower and converging at higher frequencies of conductivity spectra with increased temperature. With increasing temperature, the plot shows that conductivity increments. In the low-frequency region, frequency independent conductivity behavior is noticed, but that becomes sensitive in the high-frequency region, generally known as hopping frequency, moved to the higher-frequency side with increment of temperature. The conductivity increments in the higher-frequency region, because of the hopping of charge carriers in finite clusters.

### Adsorption of dyes onto Zn–Fe LDH

**Figure 11:**
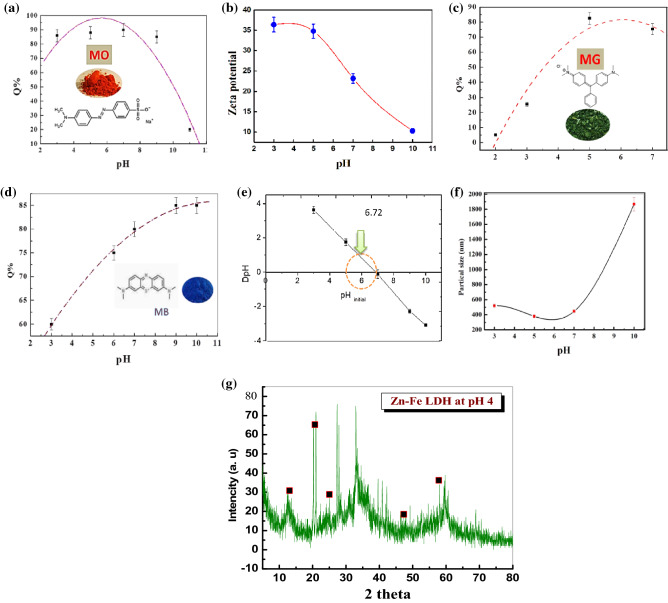
Percent removal of different dyes (**a**,**c**,**d**); (**b**) zeta potential; (**e**) PZC; (**f**) partial size distribution of Zn–Fe LDH at different pH values; and (**g**) XRD spectra of Zn–Fe LDH at pH 4.

The pH has a great effect on the adsorption process, where above a pH of 4, MO develops a negative charge owing to its dissociation constant of 3.47 (Fig. 11a). At pH 7, the removal reaches its maximum adsorption value, and beyond this value, the adsorption decreases. In acidic media, MO was protonated at its nitrogen–nitrogen double bond, and so the adsorption percent decreased owing to the forces of electrostatic repulsion. Reaching equilibrium in alkaline media was difficult owing to a high quantity of OH^–^, which competes with anionic MO and thereby prevents adsorption equilibrium^37^. Zeta potential is a technique to study the stability of the prepared material and dispersion in solution (Fig. 11b). The high stability of the Zn–Fe LDH nanoparticle dispersions is related to the high positive zeta potential under acidic medium, leading to strong particle–particle repulsions. Moreover, in considering the surface charge and surface properties for the adsorption behaviour of prepared LDH with different dyes, the positive zeta potential of LDH is consistent with the electrostatic attraction between MO and LDH and supports the process of adsorption. MG and MB removal sharply increased to 81% and 84%, respectively, at a pH of 6 for MG and 9 for MB (Fig. 11c,d). At a pH higher than 6–8, the adsorption of MG likely increases owing to OH^−^ groups that are attracted to the positive molecules of MG. It was reported that the PZC value of Zn–Fe LDH is 6.72 (Fig. 11e)^23^, which is consistent with electrostatic attraction either between MG molecules or between MB and Zn–Fe LDH.

To demonstrate the stability of the partial in several media, we estimate the practical size distribution (DLS) measurements. As observed in Fig. 11f, using Zn–Fe LDH with a smaller hydrodynamic size led to better aquatic stability and higher zeta potential. Moreover, this could allow its long-term application as a potential adsorbent for different pollutants in aquatic systems. Furthermore, to prove the stability of the adsorbent at low pH, we investigated the XRD spectrum of LDH in acidic media (Fig. 11g). The results showed that the material was maintained at characteristic peaks of LDH^34^, as presented in Fig. 11g.

#### Adsorption isotherm studies

**Figure 12:**
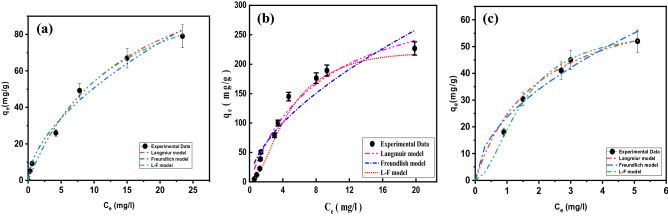
Experimental adsorption isotherm data of (**a**) MB, (**b**) MO, and (**c**) MG dye on LDH fitted using two- and three-parameter isotherms.

Adsorption isotherms explain how molecules of the adsorbate are distributed between the solid and liquid phases as the adsorption process reaches an equilibrium state. Modeling is crucial to comparing and predicting the LDH for which two- or three-parameter isotherm models apply well. Two-parameter models are commonly applied owing to simplicity and ease of fitting, and because the two-parameter models fit the data well, the use of a more complex model is not required. The adsorption isotherms for MB, MO, and MG are shown in Fig. 12.

Isotherm models explain the behavior of the adsorption of MB, MO, and MG well upon comparing the calculated values from adsorption isotherms with experimental values applied to fit the experimental data using a nonlinear relationship with a Langmuir adsorption isotherm model^38^. The Langmuir adsorption isotherm is widely used for the modeling of homogeneous adsorption on the surface of the monolayer and assumes that the adsorbent surface is uniform and that all sorption sites are identical. The Freundlich isotherm model is suitable for heterogeneous adsorbent surfaces and multilayer adsorption. The Langmuir–Freundlich (L–F) isotherm model is used for both heterogeneous and homogeneous distributions at high and low concentrations^39^. Table 1 shows the adsorption behavior of MB, MO, and MG well based on the statistical analysis of the correlation coefficient R^2^; for MB, R^2^ was 0.996 and 0.993 for the Langmuir and Langmuir–Freundlich isotherm models, respectively, whereas the *q*_*e*_ was 133.29 mg/g. Based upon this result, the Langmuir model was the best model for explaining the adsorption process, where homogeneous adsorption is on the surface of the monolayer, and the surface of LDH is uniform and without interactions between adsorbents. This indicates that the Langmuir model is more suitable for explaining the process of MB adsorption and better represents the experimental data (Fig. S4). The R^2^ for MO was 0.990 for the Langmuir–Freundlich model isotherm, and the following order for R^2^ was observed: Langmuir–Freundlich > Langmuir > Freundlich. The maximum adsorptivity (*q*_*e*_) was 230.68, and these results indicate that multilayer adsorption occurred on heterogeneous surfaces. The Langmuir–Freundlich model was more suitable to describe and explain the process of the adsorption of MO with Zn–Fe LDH. For MG, the correlation coefficient was 0.997 for the Langmuir–Freundlich model isotherm. This suggests that the Langmuir–Freundlich model was better than other applied models due to the presence of chemical bonds between metal ions (LDH) and dye, with ion exchange in solution. (Langmuir–Freundlich > Freundlich > Langmuir; *q*_*e*_ of 57.34 mg/g.).

**Table 1:** Adsorption isotherm constants for the adsorption of MB, MO, and MG dyes in single systems.

Isotherm models	Adjustable model parameters	Values	*R*^2^	χ^2^
**Methylene blue**				
Langmuir	*q*_*max*_	133.29	0.996	0.00035
	*K*_ad_	0.069		
Freundlich	*K*_f_	13.48	0.980	0.00120
	1/*n*_F_	0.575		
Langmuir–Freundlich	*q*_*max*_	119.51	0.993	0.00038
	*K*_LF_	0.0836		
	β_*LF*_	1.04		
**Methyl orange**				
Langmuir	*q*_*max*_	508.20	0.996	0.00035
	*K*_ad_	0.069		
Freundlich	*K*_f_	13.48	0.980	0.00120
	1/*n*_F_	0.575		
Langmuir–Freundlich	*q*_*max*_	230.68	0.990	0.000629
	*K*_LF_	0.220		
	β_*LF*_	1.86		
**Malachite Green**				
Langmuir	*q*_*max*_	71.74	0.969	0.00029
	*K*_ad_	0.527		
Freundlich	*K*_f_	23.63	0.917	0.00180
	1/*n*_F_	0.526		
Langmuir–Freundlich	*q*_*max*_	57.34	0.997	0.000052
	*K*_LF_	0.685		
	β_*LF*_	1.854		

**Figure 13:**
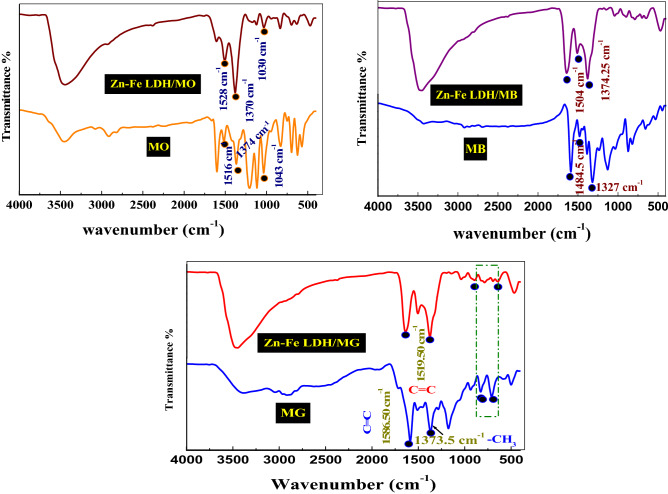
The FT-IR spectra of Zn–Fe LDH samples before and after adsorption of MG, MB, and MO dyes.

The mechanism of adsorption of dye on the LDH surface can be investigated using FT-IR spectra. The FT-IR spectrum of the Zn–Fe LDH, after the addition of MG, showed peaks in the 800–400 cm^−1^ fingerprint wavenumber region. These peaks are consistent with the di-substituted and monosubstituted benzene rings present in MG and confirm its adsorption onto the LDH surface (Fig. 12). This was further supported by the characteristic peak at 1585 cm^−1^ related to the C=C of the benzene ring as well as the peak at 1373.5 cm^−1^ owing to –CH_3_. The adsorption of MO or MB on Zn–Fe LDH was confirmed through FT-IR analysis (Fig. 13). We observe a small intense peak at 1618 cm^−1^ consistent with the C–C vibration band of the benzene ring related to the MO chemical structure. The peaks at 1138 and 1370 cm^−1^ were related to stretching vibrations of C–C and C–N, respectively. C–H stretching vibration peaks of the benzene ring were located at 1030 and 837 cm^−1^, whereas the peaks present at 629.5 cm^−1^ were assigned to the C–S stretching vibrations. Hence, we can conclude that MO, MB or MG is adsorbed to the surface of Zn–Fe LDH. Also, the basal spacing of the (003) plane decreased from 0.414 nm in the case of LDH to 0.6933, 0.693 and 0.8990 in LDH/MG, LDH/MB and LDH/MO respectively, which revealed a high effective penetration of dye into LDH interlayers^40^. This increasing may refer to one of the following reasons: the anion exchange of nitrate molecules, rearrangement of Zn–Fe LDH ions, and removal of water molecules or the adsorption of dye molecules on the surface of LDH via hydrogen-bonding, as per the scheme submitted^41^. Table 2 shows the comparison of Zn–Fe LDH with other adsorbents to further estimate the role of synthesis materials for the wastewater remediation of anionic dyes (MO) and cationic dyes (MB and MG). The maximum adsorption capacity (*q*_*max*_, obtained from isotherm model fits) for this LDH is carefully compared with those for other adsorbents. Considering the high adsorption capacity, it seems that the Zn–Fe LDH prepared in this study could potentially be used as a cost-effective adsorbent for dye-polluted aquatic systems.

**Table 2:** Comparison of the maximum adsorption capacities for the removal of MO, MB or MG using different LDHs as adsorbents.

Adsorbents	*q*_max_ (mg/g)	C initial (mg/L)	Dye/References
Cu/Cr-LDH	55.86	50	MG/^42^
MMT@NiFe LDH	99.18	30	MB/^43^
Fe/SCD-LDH	83.44	100	MB/^44^
Mg_3_Al LDH	46.72	0.75	MB/^45^
C-LDH	41.97	8.00	MB/^46^
C-LDH	48.83	32.70	MO/^46^
Mg-Fe LDH	71.99	10.00	MB/^47^
Mn-Fe LDH	65.78	10.00	MB/^47^
Zn–Fe LDH	133.29/MB	20	This study
	508.20/MO		
	71.74/MG		

#### Ternary adsorption isotherm studies

**Figure 14:**
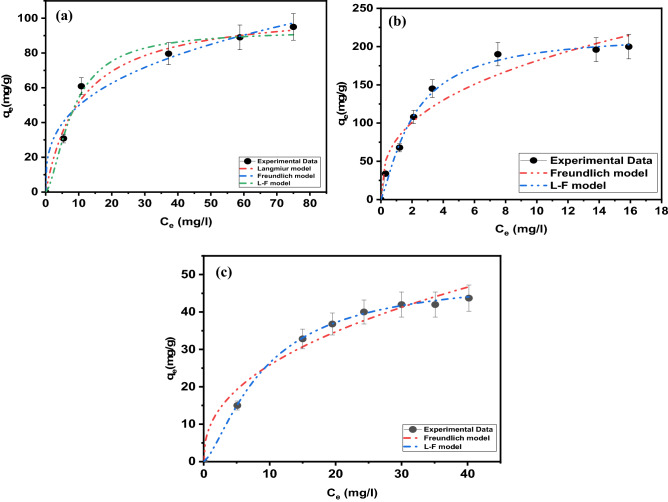
Experimental adsorption isotherm data from the ternary system for (**a**) MB and (**b**) MO and MG dyes on the LDH fitted using the two-parameter isotherm.

As shown in Fig. 14, the adsorption capacity (*q*_*e*_) of dyes is negatively affected when the concentration of each dye in the mixture is increased in the range of 10–1000 mg/L. The decrease in the adsorption capacity of MB and MG is lower than MO, which is probably due to the affinity of MO toward the positively charge adsorbent surface (Table 3). On the other hand, MG and MB show the opposite behavior from that shown by MO since Zn–Fe LDH presents a high affinity toward anionic dyes (Fig. S5). We can conclude that interactions are being favored basically for the removal of anionic dyes like MO rather than cationic, as is the case for MB or MG. Langmuir, Freundlich, and Langmuir–Freundlich adsorption isotherms are applied to study the adsorption capacity exhibited by LDH used and the concentration of dyes at equilibrium. The Langmuir adsorption isotherm assumes homogeneous monolayer adsorption, whereas the Freundlich isotherm assumes heterogeneous multilayer adsorption. The adsorption isotherms assist in investigating the maximum adsorption capacity and the adsorption mechanism (Table 3). As shown from the calculated parameters (Table 3) and the isotherm plots for dye adsorption (Fig. 14), the Langmuir–Freundlich isotherm model better explains dye adsorption on LDH, indicating multilayer adsorption in the following order: MO > MB > MG. MB and MO are flat and slightly hydrophobic molecules with rigid heterocyclic aromatic rings^48^. Hydrophobic interactions lead to a strong tendency of MO and MB to form dimers and trimers (the dimerization of MO and MB were 3.96 and 2.38, respectively^49^). This results in multilayers of MO formed on the surface of the adsorbent^30^, which agrees with the adsorption results (Table 3)^34–37,50^.

**Table 3:** Langmuir, Freundlich, and Langmuir–Freundulich isotherm constants for the adsorption on of MB, MB, and MG dyes on LDH in the ternary system.

Isotherm models	Adjustable model parameters	Values	*R*^2^	χ^2^
**Methylene blue**				
Langmuir	*q*_*max*_	106.32	0.965	0.00075
	*K*_ad_	0.0939		
Freundlich	*K*_f_	23.093	0.923	0.00164
	1/*n*_F_	0.333		
Langmuir–Freundlich	*q*_*max*_	93.122	0.973	0.00057
	*K*_LF_	0.1229		
	β_*LF*_	1.593		
**Methyl orange**				
Langmuir	*q*_*max*_	210.0	0.9077	0.056
	*K*_ad_	0.80		
Freundlich	*K*_f_	78.51	0.912	0.00258
	1/*n*_F_	0.364		
Langmuir–Freundlich	*q*_*max*_	217.97	0.989	0.000326
	*K*_LF_	0.4828		
	β_*LF*_	1.259		
**Malachite Green**				
Langmuir	*q*_*max*_	59.24	0.980	0.0041
	*K*_ad_	0.07		
Freundlich	*K*_f_	9.67	0.928	0.000675
	1/*n*_F_	0.426		
Langmuir–Freundlich	*q*_*max*_	49.57	0.999	0.0000076
	*K*_LF_	0.109		
	β_*LF*_	1.401		

#### Kinetic studies

The equilibrium time is considered one of the important factors that affect the cost and applicability of the adsorption process. Several experiments happened at different times to investigate the equilibrium time of the adsorption process. After that, fitting the obtained data to three kinetic models. Figure S6 shows the effect of the time on the adsorption process of MO solution (pH 7) 30 mg/L onto Zn–Fe LDH (0.01 mg/50 mL) at 30 ± 0.5 °C. We observed from Fig. S6 that the adsorption of MO happened at a fast rate during the first 20 min, then the rate of adsorption decreased until the equilibrium was achieved within 90 min. *R*^2^, we found the pseudo-first order, pseudo-second order and Avirma were the best fit kinetic models with *R*^2^ = 0.997 (Table 4).

**Table 4:** Kinetic model parameters (MO^+^: 30 mg/L; Zn–Fe LDH: 0.01 mg/50 mL).

Kinetic models	Parameters	Values
Pseudo first order	*K*_1_	0.018
	q_e_ Exp (mg/g)	34.90
	q_e_ Calc (mg/g)	34.24
	R^2^	0.997
Pseudo second order	*K*_2_	1.03
	q_e_ Exp (mg/g)	34.90
	q_e_ Calc (mg/g)	34.24
	R^2^	0.997
Avrami	q_e_ Exp (mg/g)	34.90
	q_e_ Calc (mg/g)	34.24
	K_av_	1.814
	*n*_av_	1.714
	R^2^	0.997

#### Photocatalytic degradation

**Figure 15:**
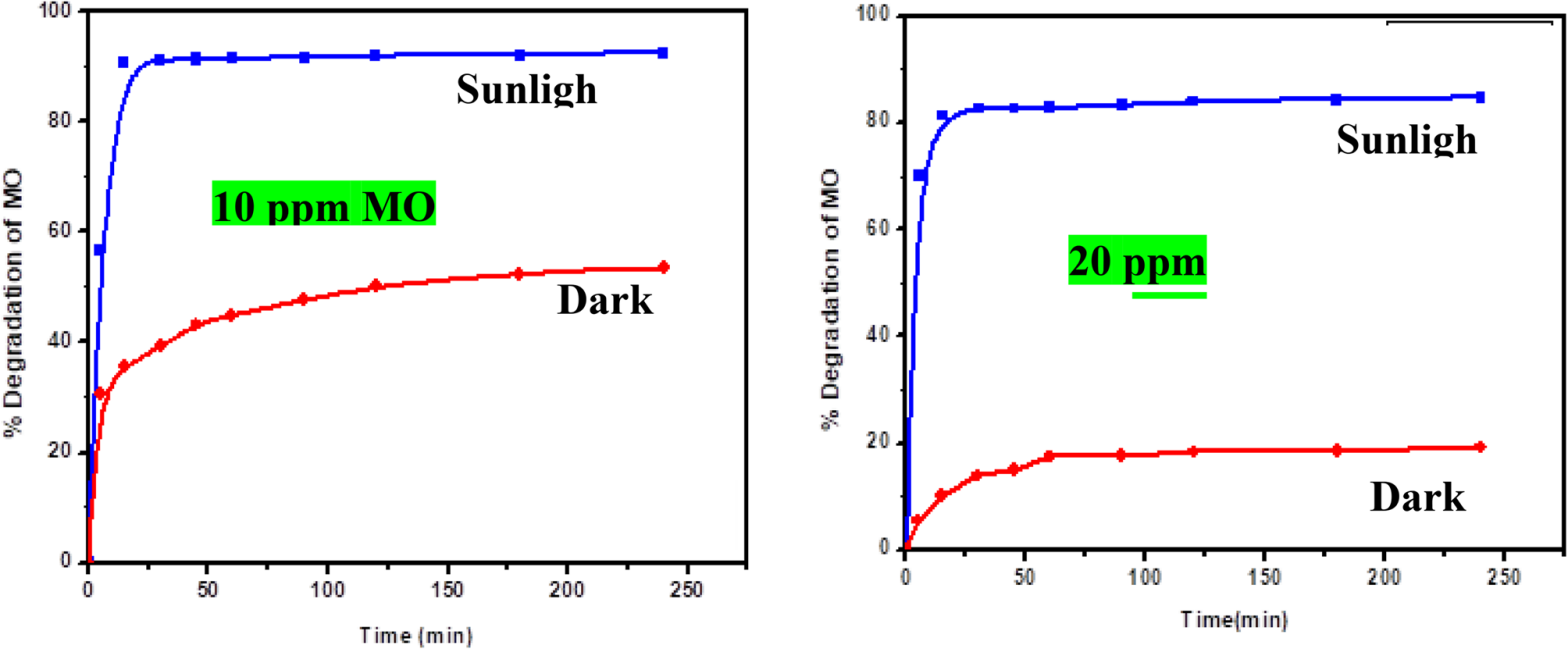
influence of the sunlight irradiations on the degradation efficiency for MO [10 and 20 ppm, respectively] over Zn–Fe LDH at different irradiation intervals.

As we all recognize, light ray absorption by the photocatalyst, the separation of the photoelectrons, and holes are important factors during the photocatalytic interaction. According to the above experimental result data, the proposed photodegradation mechanism of Zn–Fe LDH can be illustrated that LDH can absorb visible light rays because of the narrow band gap of 1.765 eV. Under solar light irradiation, the electrons in the valence band of LDH can be inspired to the conduction band, leaving holes in the valence band. The structure of LDH can effectively restrain the recombination of photoelectrons and holes to improve photocatalytic activity. The holes left in the valence band of LDH can more easily induce the formation of hydroxyl radicals (·OH) from OH groups^51^ absorbed on the surface. Besides, the electrons passed by LDH are scavenged by the absorbed molecular oxygen (O_2_) to form O_2_^−^ radicals. These radical groups of ·OH and O_2_^−^ will result in the decomposition of MO. The effect of initial MO concentration on photodegradation efficiency has been achieved by varying the initial MO concentration between 10 and 20 mg L^−1^ with other parameters such as catalyst concentration, reaction temperature, and pH value remaining constant, and the result is shown in Fig. 15. It could be shown that the photodegradation efficiency decreases with an increase in the initial concentration of MO. The assumed reason is that equilibrium adsorption of reactants on the surface sites of the catalyst increases with the MO concentration, and with the increase of the initial concentration of MO, the dye molecules absorb the light much more than the catalyst does, which is thought to have an inhibitive effect on the photodegradation process, and so the rate of photocatalytic reaction decreases^52^.

### Monte Carlo (MC) simulation

**Figure 16:**
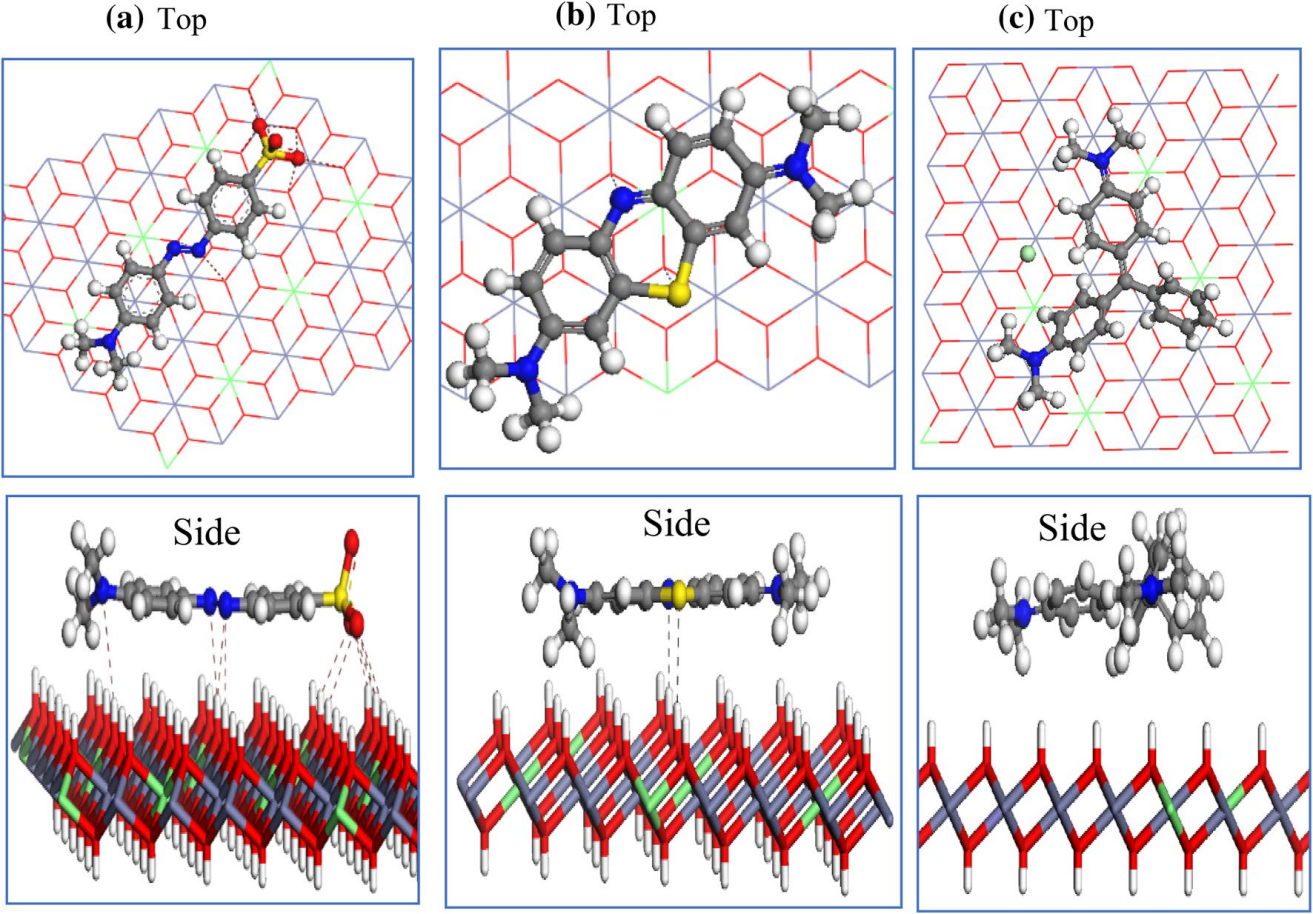
The adsorption of MO (**a**), MB (**b**), and MG (**c**) molecules on the Zn–Fe LDH (001) surface, as obtained from the MC simulation.

**Figure 17:**
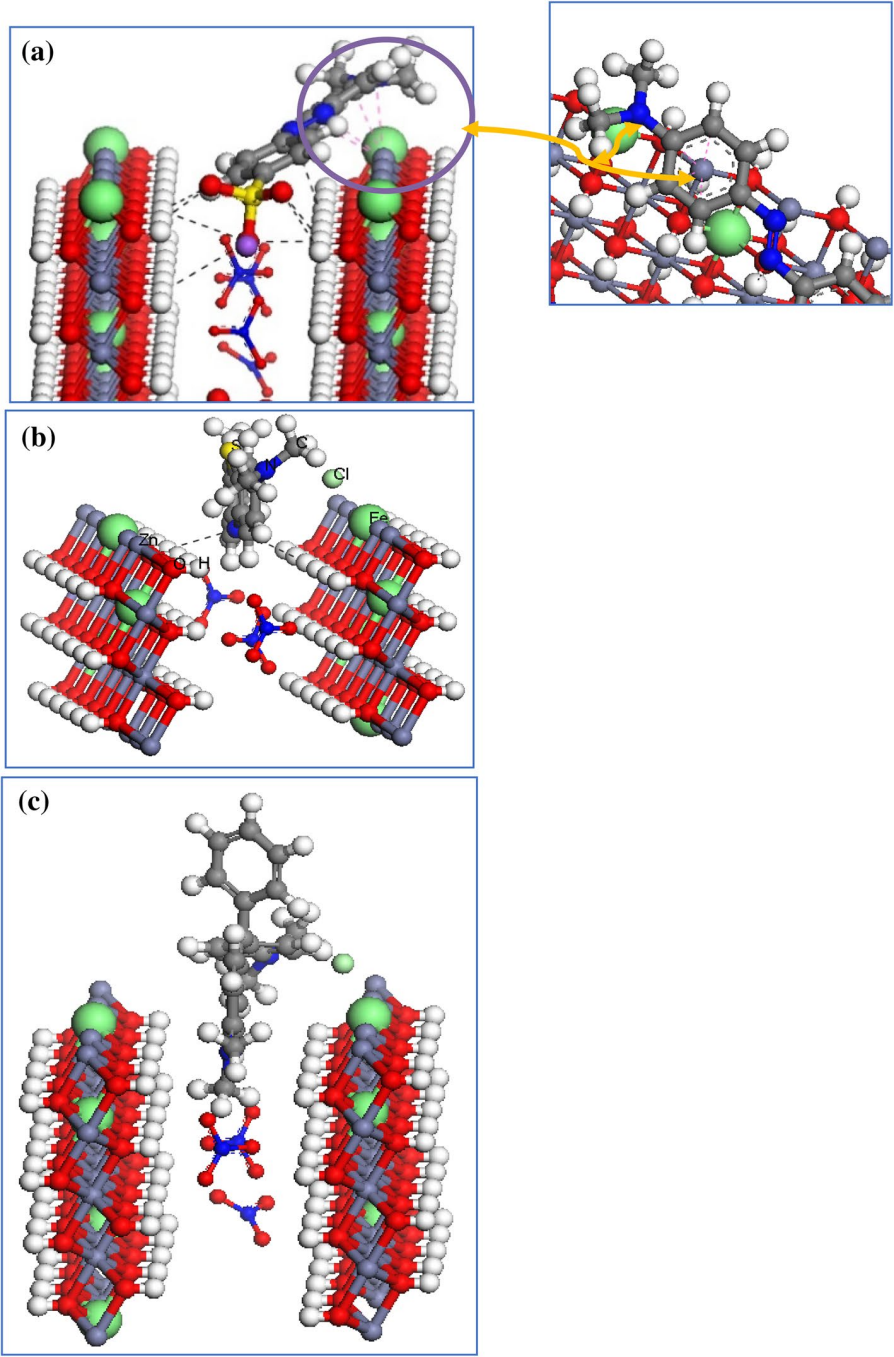
The adsorption of MO (**a**), MB (**b**), and MG (**c**) molecules on the Zn–Fe LDH (010) surface, as obtained from the MC simulation.

To understand the interactions between the dyes and LDH surface, MC studies were performed using Zn–Fe LDH (001) and (010) planes. The lowest-energy structures between the studied adsorbates (MO, MB, and MG) and the Zn–Fe LDH (001) and (010) surfaces as obtained from the MC simulations are shown in Figs. 15 and 16. The studied dyes consist of hydrogen-bonding acceptor atoms (N, O, and S atoms). Therefore, they may form either intermolecular hydrogen bonds (HBs) with the hydroxyl hydrogen atoms on the LDH surface, or coordinate bonds with the Zn or Fe actions. The former interactions can be explored by using the (001) surface, while the latter interactions can be viewed by using the (010) model, respectively. As shown in Fig. 16, the hydrogen-bonding acceptor atoms of MO and MB formed intermolecular HBs with the hydroxyl hydrogen atoms on the (001) surface. The MO molecule formed the HBs through the sulfonic oxygen (two oxygens out of the three oxygens) and the three nitrogen atoms. While the MB molecule formed HBs with the LDH hydroxyl groups via the aromatic sulphur and nitrogen atoms. In the case of MG, there is no HBs observed. The adsorption of MO molecule on the LDH (010) surface displayed that the benzene-1-sulfonate moiety was located between two LDH layers, as given in Fig. 17a, and sulfonic oxygens were formed HBs with the hydroxyl hydrogens of both layers. A diazinyl-moiety nitrogen atom was found to form HB with the hydroxyl hydrogen of one LDH layer. A cation–π interaction was observed between Zn cation and the phenyl group as shown in that figure. Additionally, the nitrogen atom of the dimethylamino group was found to be located at a distance of 2.51 Å from a Fe atom, indicating a coordination bond might be formed between them. In the case of MB, the aromatic nitrogen formed HBs with hydroxyl hydrogen of two LDH layers, as shown in Fig. 17b. It was found that the MG does not form hydrogen bonds with the LDH (010) surface (Fig. 17c). The adsorption energies of MO, MB, and MG on the (001) Zn–Fe LDH surface were − 126.8, − 80.25, and − 79.2 kcal mol^−1^, and on the (010) Zn–Fe LDH surface were − 140.2, − 100.4, and − 96.7 kcal·mol^−1^, respectively. The electrostatic interaction of MO, MB, and MG with the (001) Zn–Fe LDH surface were, − 4.51, − 3.96, and − 3.63 kcal·mol^−1^, while with (010) Zn–Fe LDH surface were, − 33.39, − 27.27, and − 25.43 kcal·mol^−1^, respectively. This obtained trend in both adsorption energies and electrostatic interactions agrees with the experimental adsorption capacities of the studied dyes on the Zn–Fe LDH.

## Conclusions

In this research, a coprecipitation method was applied to synthesise Zn–Fe-LDH, then the Zn–Fe-LDH was used for dye adsorption in single and ternary systems after investigating the structure of the prepared material using physical and chemical methods. For the single system, the maximum adsorption capacities were 230.68, 133.29, and 57.34 mg/g for MO, MB, and MG, respectively; for the ternary solution, the respective values were 217.97, 93.122, and 49.57 mg/g. Experimental isotherm data fits well with nonlinear isotherm models. Furthermore, pseudo-first-order, pseudo-second-order, and Avrami models described the adsorption kinetic data for MO, demonstrating chemisorption and physisorption properties. The optimum pH was 7, 9, and 6 for MO, MB, and MG, respectively. The adsorption mechanisms were investigated for dyes through XRD and FT-IR analyses and Monte Carlo simulation. Moreover, LDH proved that it could be applied as a photocatalyst for dye-polluted water.

## Supplementary Information

Supplementary Information.
