# Supplementary material for: Understanding the physicochemical properties of Zn–Fe LDH nanostructure as sorbent material for removing of anionic and cationic dyes mixture
Source: Sci Rep. 2021 Nov 1;11:21365. doi: 10.1038/s41598-021-00437-w (PMC8560778; doi:10.1038/s41598-021-00437-w)
Supplement: Supplementary file 1 — Supplementary Information. [file 41598_2021_437_MOESM1_ESM.docx]

**Understanding the Physicochemical properties of Zn-Fe LDH Nanostructure as sorbent material for removing of anionic and cationic Dyes mixture**

Rehab K. Mahmoud^a,^*, Mohamed Taha^b^, Amal Zaher^c^ , Rafat M. Amin^d,^*

*^a^Department of Chemistry, Faculty of Science, Beni-Suef University, 62511 Beni-Suef, Egypt*

*^b^Materials Science and Nanotechnology Department, Faculty of Postgraduate Studies for Advanced Sciences (PSAS), Beni-Suef University, Beni-Suef, Egypt*

^c^*Environmental Science and Industrial Development Department, Faculty of Postgraduate studies for Advanced Sciences, Beni-Suef University, Beni-Suef, Egypt*

*^d^Department of Physics, Faculty of Science, Beni-Suef University, 62511 Beni-Suef, Egypt*

**Table S 1** Chemicals and physical characteristics of methylene blue, methyl orange and malachite green.

| Malachite green [23] | Methyl orange [24,25] | Methelene blue [26] | Dye name |
| --- | --- | --- | --- |
| Green crystalline powder | Orange-yellow powder | Dark green crystals or powder | Appearance |
| C_23_H_25_ClN_2_ | C_14_H_14_N_3_NaO_3_S | C_16_H1_8_N_3_SCl | Molecular formula |
| 364.9 | 327.33 | 319.85 | Molecular weight (g/mol) |
| 1.044 | 1.28 | 0.98 | Density (g/cm^3^) |
| 616.9 | 464 | 660 | λ max (nm) |
| Cationic | Anionic | Cationic | \| Type of dye \| \| --- \| |

**Table S2.** The adsorption isotherm and kinetics models

| Isotherm models | Equations | Kinetics models | Equations |
| --- | --- | --- | --- |
| Langmuir |  | Pseudo-first-order |  |
| Freundlich |  | Pseudo-second-order |  |
| Langmuir–Freundlich | 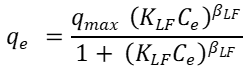 |  |  |
|  |  | Intraparticle diffusion  Avrami |    |

* The adjustable isotherm/kinetics model parameters are defined in the list of symbols


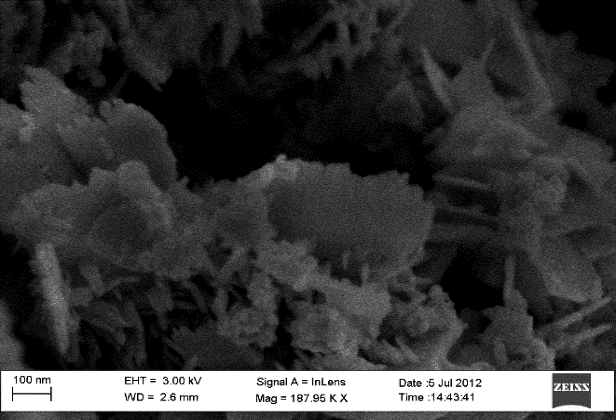

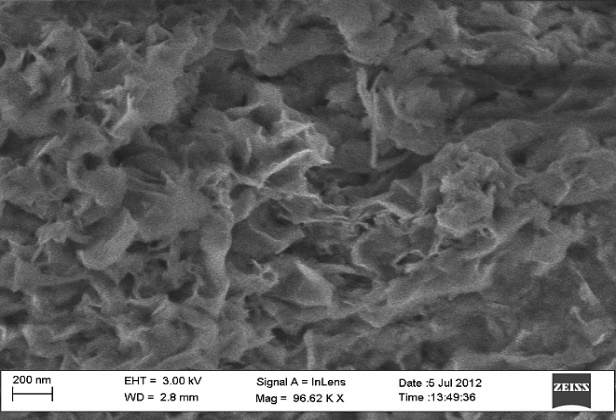

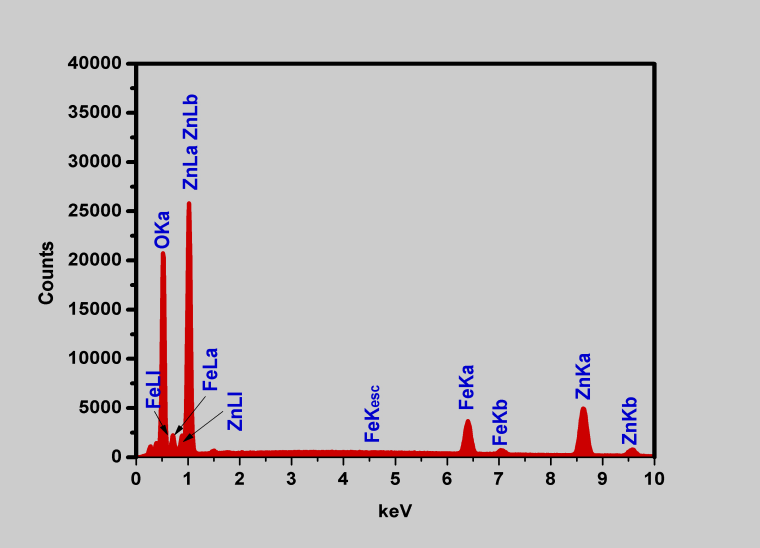

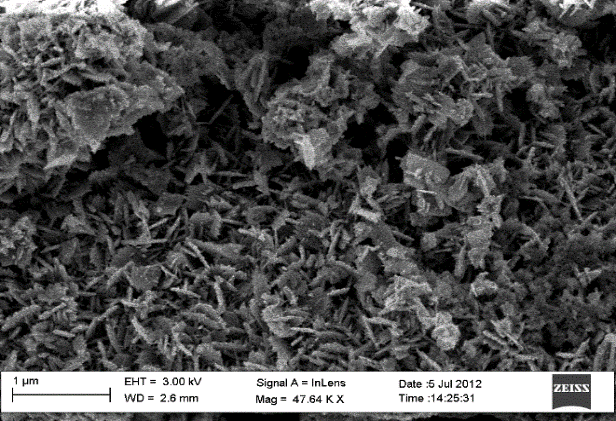


**(a)**

**(b)**

**(c)**


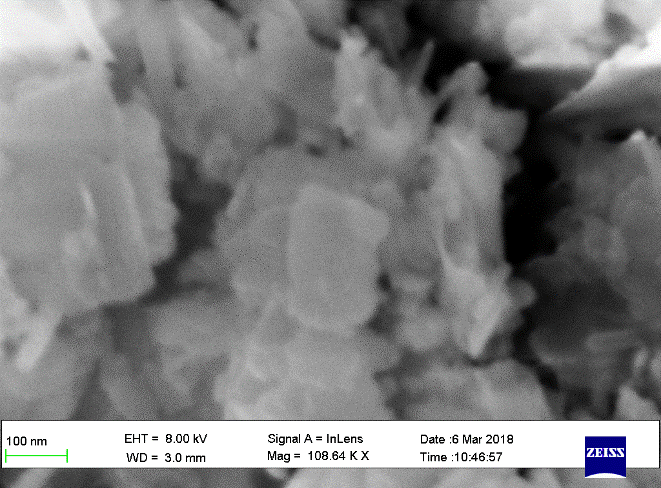


**(d)**

**Fig. S1.** EDX (a ) and FESEM images of the prepared Zn-Fe LDH (b-d)


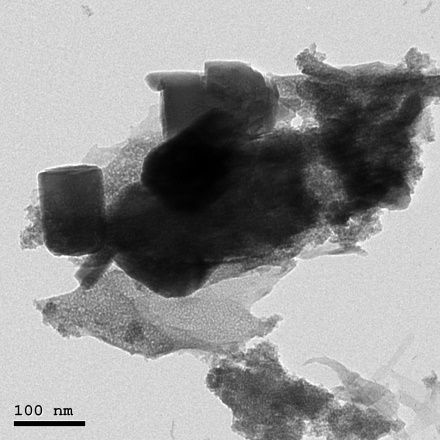


**(a)**


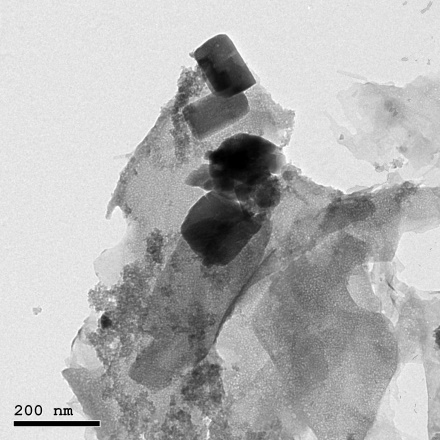


**(b)**


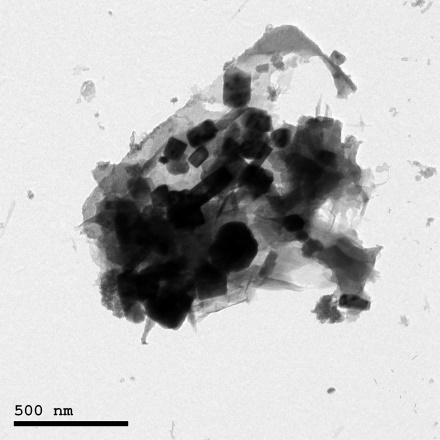


**(d)**


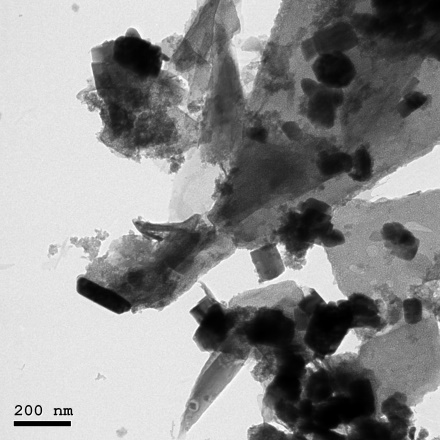


**(c)**


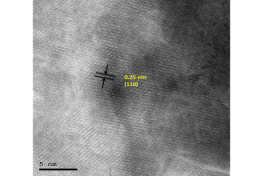

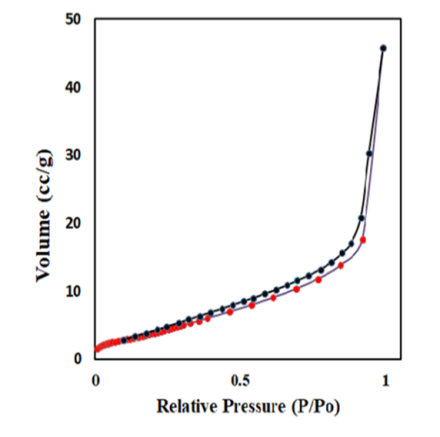

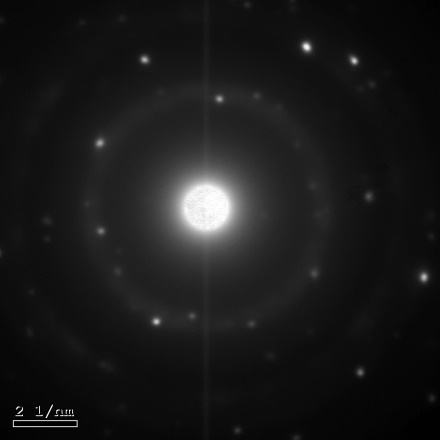


**(e)**

**(f)**

Hexagonal

**Layer**

**Layer rolling**

**(g)**


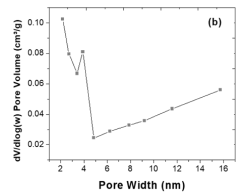


**h))**

**Fig. S2.** HRTEM images of the (a-d); inset figure represent interplanar spacing (g); (SAED) The Selected Area Electron Diffraction (e) N_2_ adsorption-desorption isotherms (f) and inset Figure (h) for pore volume distribution for the prepared Zn-Fe LDH.

Transmittance (%)

(a)

(b)


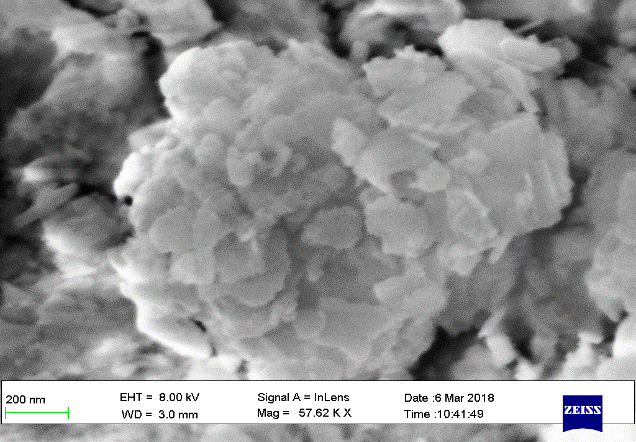


**(c )**

**Fig. S3.** (a) The FT-IR spectra, (b) XRD patterns, and (c) FESEM of the prepared Zn–Fe LDH.


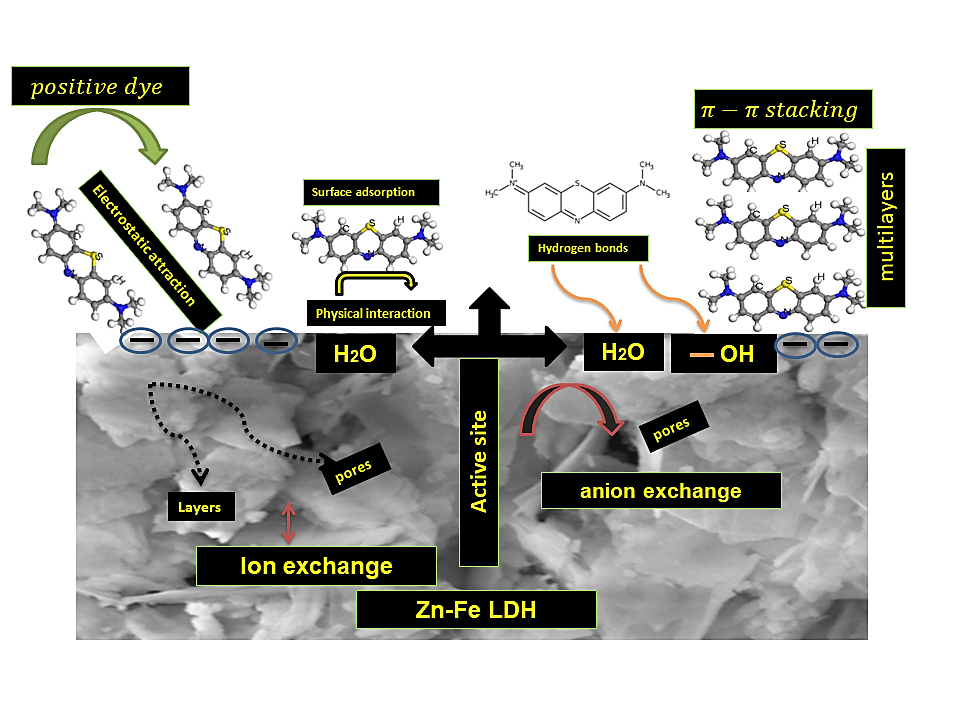


**Fig.S4** MB adsorption mechanisms onto the Zn-Fe LDH

**Fig. S5**. UV-Vis Spectra of the ternary systems (MO, MB and MG) 20 mg L^-1^ of each at 0.02 g Zn-Fe LDH, pH ~7, v=20 ml and T=25 C^o^

**Fig. S6** shows the effect of the time on the adsorption process of MO solution (pH 7) 30 mg/L onto Zn–Fe LDH (0.01 mg/50 ml) at 30 ± 0.5 °C
